# Towards a constructionist approach to emotions: verification of the three-dimensional model of affect with EEG-independent component analysis

**DOI:** 10.1007/s00221-014-4149-9

**Published:** 2014-11-26

**Authors:** Miroslaw Wyczesany, Tomasz S. Ligeza

**Affiliations:** Psychophysiology Laboratory, Institute of Psychology, Jagiellonian University, Ingardena 6, 30060 Kraków, Poland

**Keywords:** Emotions, Emotional dimensions, Constructionist theory, EEG, Independent component clustering

## Abstract

The locationist model of affect, which assumes separate brain structures devoted to particular discrete emotions, is currently being questioned as it has not received enough convincing experimental support. An alternative, constructionist approach suggests that our emotional states emerge from the interaction between brain functional networks, which are related to more general, continuous affective categories. In the study, we tested whether the three-dimensional model of affect based on valence, arousal, and dominance (VAD) can reflect brain activity in a more coherent way than the traditional locationist approach. Independent components of brain activity were derived from spontaneous EEG recordings and localized using the DIPFIT method. The correspondence between the spectral power of the revealed brain sources and a mood self-report quantified on the VAD space was analysed. Activation of four (out of nine) clusters of independent brain sources could be successfully explained by the specific combination of three VAD dimensions. The results support the constructionist theory of emotions.

## Introduction

The question of which specific parts of the human brain “produce” emotions is a fundamental one for human neuroscience. The answer has not yet been found, and difficulties lie in the complex nature of emotions, which are comprised of multiple components such as autonomic arousal, behaviour, and subjective experience. These aspects are known to be dependent on partly separate neural systems. In this paper, we focus on neural systems underlying phenomenological aspects of affective states, which bring valence “colours” to our everyday life experience. Co-variation of EEG data with the ongoing emotional state is considered in the context of two dominant literature models: the “locationist” and the “constructionist” approach.

The search for a neural basis of affective experience has a long history. During this time, the locationist paradigm (closely linked with the basic emotions theory) has become the most prevalent approach. It assumes that discrete emotions (such as sadness, happiness, and fear) are “created” by specific and usually separate brain centres (Ekman 1992; Barrett 2006a). It is then supposed that: (1) a particular brain region shows increased activity each time a person experiences a particular emotion (consistency); (2) every discrete emotion has a unique neural pattern (specificity). For example, emotions of fear and sadness are proposed to be mediated by distinct neural structures, and each occurrence of a particular emotion is associated with the same brain arousal pattern.

Several meta-analytic reviews have been conducted in a locationist paradigm. Two influential fMRI and PET reviews (Phan et al. 2002; Murphy et al. 2003) were generally inconclusive and only partially supportive of the view that basic emotions are uniquely represented in specific brain regions. Both reviews focused on five basics emotions: happiness, sadness, anger, fear, and disgust. They managed to find some common neural activations associated with emotions of fear and sadness and concluded that the underlying neural systems of basic emotions are only partly separate. What is more, the reviews reached only incomplete agreement regarding the associations between specific brain structures and particular basic emotions. More recent fMRI meta-analyses (Kober et al. 2008; Lindquist et al. 2012) have challenged the usefulness of a locationist approach in explaining brain bases of emotions. The main criticism is that discrete emotions are not consistently and specifically associated with activation of a particular brain structure (Posner et al. 2005; Barrett et al. 2007). In addition, the question arises as to whether discrete emotional states really exist as separate brain and body states or whether they are just reflecting emotion words available in natural languages, dependent on one’s interpretation of the situational context (Schachter and Singer 1962; Russell 2003; Barrett 2006b). All things considered, the fundamental assumptions of a locationist approach have not been confirmed.

Recently, the constructionist approach has been introduced to analyse the neural basis of emotions from a perspective of broader, less-specific emotional dimensions. It assumes that elicitation of emotions involves the interaction of wider neural networks, which subserve domain-general functions that are not specific to single discrete emotions (Lindquist and Barrett 2012; Barrett and Satpute 2013; Lindquist 2013). As there is some recent evidence that focusing on more general emotional dimensions instead of discrete affective categories may enable easier interpretation of neuroimaging data, we follow this view in the present study. Therefore, we propose a dimensional approach as a basis for the search for neural correlates of emotional experience. The dimensional concept is well established in psychometric studies of emotions and originates from factor analyses of self-report questionnaire data (Russell et al. 1981; Watson and Tellegen 1985). It posits that all emotions can be represented by a small number of dimensions and aims to provide the most economical and synthetic framework for a description of the affective state. However, there is substantial disagreement about the optimal number of dimensions to characterise emotions. Most of the models propose two factors: valence (ranging from negative to positive) and arousal (low to high; Posner et al. 2005). Nonetheless, the two-dimensional models have been criticized for their lack of differentiation when it comes to emotions that are close on the valence and arousal space, such as anger and fear (Tellegen et al. 1999; Fontaine et al. 2007). Therefore, we propose to use a well-established, three-dimensional space with the additional factor of dominance, representing a sense of control while experiencing the emotion and its dominant nature (high dominance can be considered as an external, while low dominance as an internal locus of control; Mehrabian 1970; Russell and Mehrabian 1977; Bradley and Lang 1994). It is commonly referred to as the valence (or pleasure)–arousal–dominance (VAD) model. As the dominance dimension is coupled with the relationship between the person and their environment, it is also linked to a threat–challenge dimension (Lazarus 1966; Blascovich and Tomaka 1996), whereby one may feel in control of a given situation and perceive it as a challenge, or, on the other hand, be overwhelmed and threatened by the situation. To sum up, the proposed VAD model seems to be sufficiently concise and, on the other hand, have enough discriminating power to precisely describe the emotional experience.

Although there are a considerable number of neuroimaging studies regarding valence or arousal dimensions, the issue still remains elusive. The search for neural bases of emotional dimensions dates back to studies where the concept of frontal asymmetry was investigated in terms of the state of valence using EEG (Davidson et al. 1979). Further development of neuroimaging methods brought new opportunities for studying the effects of emotional valence (Lane et al. 1997). Since then, many studies have been conducted on valence or arousal dimensions, but the results remain partly inconsistent. As shown by fMRI/PET meta-analyses, no specific brain region associated with these dimensions has been found (Wager et al. 2003; Murphy et al. 2003). Some data point to the fact that the activity of a specific part of the brain could be related not only to the intensity of valence and arousal, but also to specific combinations of these two (e.g. negative and low arousal; Nielen et al. 2009). Other data suggest that the above dimensions are not reflected in activations of focal brain regions but rather in distributed patterns of activity in the whole brain (Baucom et al. 2012). Despite these ambiguities, Kassam et al. (2013) concluded that the dimensional approach (consisting of a few basic factors) is more informative with respect to understanding the neural basis of emotions than the discrete emotions account. Only one discrete emotion category (disgust) was reliably associated with focal brain activations, while basic emotional dimensions successfully denoted neural patterns of activations. This seems a promising approach and the issue is worth exploring.

Usually, in the above-mentioned studies, the researchers did not apply any self-report measurements to assess and verify the experiential aspect of emotion. Instead, participants were only instructed to watch emotional stimuli, recollect affective events, or deliberately evoke emotions. To our knowledge, only Colibazzi et al. (2010) have used self-report measures when exploring the brain basis of valence and arousal dimensions. Moreover, none of the studies have measured spontaneously experienced affect, but have rather applied mood induction procedures, which were assumed to induce the intended emotional state. The most common procedure is to present normative emotional stimuli from the International Affective Picture System (IAPS; Lang et al. 1999). Although most of the neuroimaging studies on emotions use visual stimuli to evoke specific affect, everyday emotional feelings depend on a wider class of emotional events, including stimuli of different modalities (Baumgartner et al. 2006). Unarguably, mood manipulation benefits from the fact that the affect is better controlled, but at the same time other possible confounds can be introduced, since any cognitive task performed on affective stimuli may change neural responses in regions associated with emotions (Taylor et al. 2003). Even passive watching conditions introduce additional involvement of perceptual processes, which overlap with the brain activity related to spontaneous mood experience (Garrett and Maddock 2006). The importance of more natural experimental settings with spontaneous mood measurement has recently been raised in the literature (Silvers et al. 2014). Hence, to eliminate possible confounds, we decided to focus on spontaneous affect and record brain activity in task-free conditions. On the one hand, this introduces some discretion for subjects which remains beyond experimental control. On the other, we know that in such a situation, subjects tend to focus on self-related memories, future plans, and current concerns, and this content remains emotionally laden (Gruberger et al. 2011; Fox et al. 2013). It has been argued that such self-referential mental activity recruits unspecific affective brain networks of affective experience (Wyczesany et al. 2014), which further justifies our approach. Finally, to our knowledge, none of the studies have recognized the neural bases of the dominance dimension, which was included in our analyses.

The aim of the present study was to determine whether the three-dimensional model of affect is reflected at the level of brain activity patterns. We assumed that three-dimensional emotional space (VAD) can better reflect the way neural processes underlie affective states. What is more, we assumed that affective self-report is crucial for explaining emotional phenomena. Our study fills the gap between older EEG experiments based on channel-derived spectral data and numerous fMRI studies, which provide excellent spatial resolution but also raise some doubts about the serious impact on emotional processes due to highly unnatural measurement conditions (Harmon-Jones and Peterson 2009).

Instead of classical analysis of channel data, we decided to identify the underlying brain sources that can be thought of as anatomically distinct cortical patches (Makeig et al. 1996). Such an approach allowed us to disentangle the contribution of individual neural generators, enabling more direct inferences about their functional significance. The processing path included data-driven separation of independent neural sources whose signals are normally mixed up in the EEG signal recorded from the scalp. To reconstruct the original signals, independent component analysis (ICA) decomposition was carried out using the criterion for maximizing the independence of resulting components (Delorme and Makeig 2004). Next, source localization was performed for these components in 3D brain space. An important difference between channel and component analysis occurs at the group level, since the sequence of components resulting from ICA reflects a decreasing contribution to the analysed signal, so they are arranged in descending order of strength. Due to individual differences in cortical folding, the resulting sequence of components is unique in each subject (in the case of classic channel analysis, there is always a correspondence between electrodes across all participants). Hence, to identify the functionally corresponding components across subjects, they need to be compared based on additional criteria (location, scalp map, and activation pattern) and then clustered together for group-level statistical analysis.

## Materials and methods

The procedure was compliant with the Helsinki Declaration and approved by the University Ethical Committee. A total of 58 volunteers (34 women; mean age 21; SD 1.8), participated in the study. All were medication free with no reported history of any neurological or psychiatric disease or any substance abuse. Before the procedure started, all of them signed a written consent form.

**Fig. 1:**
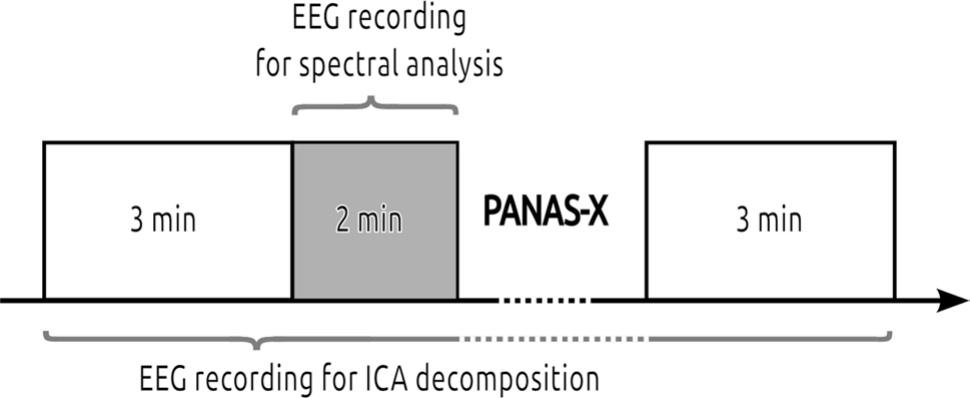
Timeline of the procedure

The experiment took place in a cabin, illuminated with dimmed light. The procedure was briefly explained to participants and electrodes were attached. The EEG recording was performed during rest conditions and there was no specific task given to participants. Instead, they were advised to sit still and keep their eyes open during the recording phase. Then, 5-min spontaneous EEG recording commenced. Immediately afterwards, subjects were presented a computerized version of the PANAS-X scale. Using 60 adjectives, subjects had to evaluate how particular adjectives referred to their current feeling at the very moment of assessment (ranging from 1—“not at all” to 5—“extremely”). Words were presented on a computer screen and subjects were supposed to answer by keyboard input. This was followed by another spontaneous EEG recording which lasted 3 min. Next, subjects took part in another procedure, which did not form part of this study. The experimental timeline is depicted in Fig. 1.

The Biosemi appliance with 64 active cap electrodes arranged according to the 10–10 system was used for EEG recording. Two additional electrodes were placed on both mastoids and another four placed above and below the right eye and in the external canthi of both eyes. Data were sampled at 256 Hz. The preprocessing was done using EEGLab package (Delorme et al. 2011). The signal was filtered with 1 Hz high-pass and 46 Hz low-pass zero-phase filters (FIR type with group delay correction, order: 846 and 74, respectively; Widmann 2012). Data channels with severe technical failures (excessive noise, prolonged loss of skin connection) were then rejected based on visual inspection (no subject had more than two channels rejected). The entire (5 min) spontaneous recording was divided into 2-s segments (overlapped 0.5 s), and an automatic artifact rejection routine was then carried out using both threshold level (below −80 μV and over +150 μV) and abnormal spectra (excessive power in beta/gamma frequencies with threshold set to 30 dB above the electrode average in the 25–45 Hz range on scalp electrodes). The lengths of the remaining data were checked (the rejection rate did not exceed 3 % of the whole recording in any given subject). Then, a blind source separation algorithm (ICA) was used to process data from scalp and ocular electrodes. Equivalent source dipoles of identified-independent components (ICs) were localized using the DIPFIT2 method (Oostenveld et al. 2011) based on a realistic standardized boundary element head model (BEM).

Artifactual ICs which originated in oculomotor activity, blinks, muscle activity, or technical problems were rejected on the basis of localization, time characteristics relative to stimuli onset and spectral power (Jung et al. 2000). Moreover, an additional check for possible confounding ocular components was applied; the normalized cross-correlation values (for zero time lag) between vertical and horizontal ocular electrodes were determined for all ICs. Two resulting values for each IC were then used as an input for clustering analysis (Ward method, number of clusters set to 2) to check the dissimilarity of ICs previously classified as ocular-related and ocular-independent.

Next, the dipoles which were localized subcortically were also ignored, due to the limited capability of surface recordings to pick up deep sources of activity together with their poor localization accuracy (Michel et al. 2004). Remaining ICs whose residual variance (RV) of dipole location was greater than 15 % were rejected (Hammon et al. 2008). Finally, the K-means clustering was applied in order to group together functionally similar ICs across subjects. The distance function for estimating the similarity included both the 3D location in standardized brain space (70 % weight) and the power spectra in the beta band (15–25 Hz; 30 % weight, reduced to 10 PCA dimensions). The former factor is relatively robust to alteration of scalp maps due to cortex folding, while the latter was introduced in order to distinguish between similarly located but functionally distinct components (Onton and Makeig 2006). The initial number of IC clusters (*k*) to be searched for was set to 20, and the threshold level for outliers was equal to 2.5 SD of the similarity distance. The clustering procedures were iterated with the *k* parameter decreased by 1 in each consecutive run until the IC clusters remained distinct in terms of anatomical and functional plausibility (Jung et al. 2007). Additionally, in cases where multiple components from one person were included in a cluster, they were averaged before the statistical analysis in order to retain the correct level of degrees of freedom (Onton et al. 2006). Since the subjects’ emotional state/activation level can slowly fluctuate, even in rest conditions, on a timescale of minutes (Wyczesany et al. 2010), the main spectral analysis was performed during the last 2 min of data recording, which directly preceded the PANAS-X questionnaire. The rationale for limiting the length of data for this analysis was to make EEG and self-report measurements quasi-simultaneous, i.e. taken in as similar an emotional state as possible (Wyczesany et al. 2008, 2009). For each IC cluster, the average of the absolute EEG spectral power in the beta range (15–25 Hz) was computed using the FFT method, and resulting values were log transformed. The beta band was chosen as a direct marker of the level of cortical activity (Andreassi 2006).

The questionnaire data were processed, yielding 11 scales which describe discrete emotional states (fear, hostility, guilt, sadness, joviality, self-assurance, attentiveness, shyness, fatigue, serenity, and surprise). Preliminarily, the Pearson-*r* correlations between the scales and beta power in all considered IC clusters were computed after ensuring a normal distribution of variables. Next, in order to convert PANAS-X scales into three-dimensional VAD reference space, we used additional data from 168 students (137 females, median age 19, range 18–43 years), who had to estimate each of the PANAS-X adjective items in terms of the three VAD dimensions. These estimates, after averaging and median split, were used to assign each of the PANAS-X scales to the category of either low or high value, separately for each VAD dimension: positive/negative valence, high/low arousal, high/low dominance (except “guilt”, which was not included in the arousal category, and “surprise”, which was not included in the dominance category, since they were ranked very close to the median value on these dimensions). The VAD scores within each high/low category were then averaged. Next, in order to determine the relationships between EEG activity and VAD dimensions, two regression analyses were carried out for each IC cluster. They included a 2 × 2 layout of PANAS-X scale categories as predictors: either valence and arousal or valence and dominance.^1^ A series of linear stepwise regressions with variance inflation factor (VIF) estimates of multicollinearity were carried out in order to determine the VAD categories configuration that can significantly predict EEG activity. Initially, all four factors were included and then automatically removed in iterative steps to retain the optimal regression model. Due to multiple comparisons performed, adjustment of resulting p-levels was carried out using the false discovery rate (FDR) procedure (Benjamini and Hochberg 1995). Additionally, we assessed the accuracy of the regression results using the leave-one-out cross-validation. Firstly, for each of the regression models, determined on n-1 samples of data, we computed residuals (differences between predicted and actual values). To aggregate these residuals into a single measure, we computed the root mean square error (RMSE). Then, the RMSE was normalized to the range of observed values (NRMSE). Finally, as the NRMSE can be considered as a percentage error (%NRMSE), we defined prediction accuracy as 100 % − %NRMSE. Apart from the main analysis based on the VAD categories, Pearson-*r* correlations between scores on original discrete PANAS-X scales and the beta power of the revealed clusters were also calculated.

## Results

Mean ratings and standard deviations for all PANAS-X scales are presented in Table 1. Translation of PANAS-X scales onto VAD dimensions and their assignment to high/low categories according to VAD values are shown in Table 2.

**Table 1:** Mean ratings and standard deviations of PANAS-X scales

	Mean	SD
Basic negative emotion scales		
Fear	1.53	0.61
Hostility	1.20	0.40
Guilt	1.43	0.64
Sadness	1.90	0.77
Basic positive emotion scales		
Joviality	2.95	0.64
Self-assurance	3.00	0.58
Attentiveness	3.80	0.57
Other affective states		
Shyness	1.69	0.60
Fatigue	2.25	0.84
Serenity	3.44	0.55
Surprise	2.11	0.83

**Table 2:** Rankings of PANAS-X scales on VAD dimensions and their assignment according to VAD values (low, moderate, high)

	Hostility	Guilt	Fear	Sadness	Shyness	Fatigue	Surprise	Calmness	Attentive	Self-assurance	Joviality
Valence											
Mean	−3.73	−3.48	−3.28	−3.26	−2.65	−2.04	0.58	2.53	3.02	3.19	4.17
SD	0.78	1.05	0.88	1.15	0.98	1.35	0.93	1.07	1.10	0.97	0.58
Assignment	NEG	NEG	NEG	NEG	NEG	NEG	POS	POS	POS	POS	POS
Arousal											
Mean	3.08	1.69	2.36	0.04	0.44	−1.00	2.62	−0.91	2.18	3.50	3.93
SD	1.12	1.76	1.15	2.16	1.76	2.18	1.28	2.06	1.56	1.03	0.77
Assignment	HI	MD	HI	LO	LO	LO	HI	LO	HI	HI	HI
Dominance											
Mean	1.81	−1.24	−0.93	−2.52	−3.10	−3.34	0.12	−1.73	2.03	3.38	2.80
SD	1.64	1.68	1.64	1.56	1.56	1.41	1.40	1.72	1.52	1.06	1.16
Assignment	HI	LO	LO	LO	LO	LO	MD	LO	HI	HI	HI

An additional eye movement check revealed two apparently separate clusters with the following values of cross-correlation: *ρ*_veog_ = 0.828 (SD 0.116), *ρ*_heog_ = 0.430 (SD 0.184) for the cluster identified as related to eye movements and *ρ*_veog_ = 0.001 (SD 0.082), *ρ*_heog_ = 0.055 (SD 0.111) for the non-oculomotor one. Thus, it was confirmed that none of the components considered in further analyses could originate from eye movements.

**Fig. 2:**
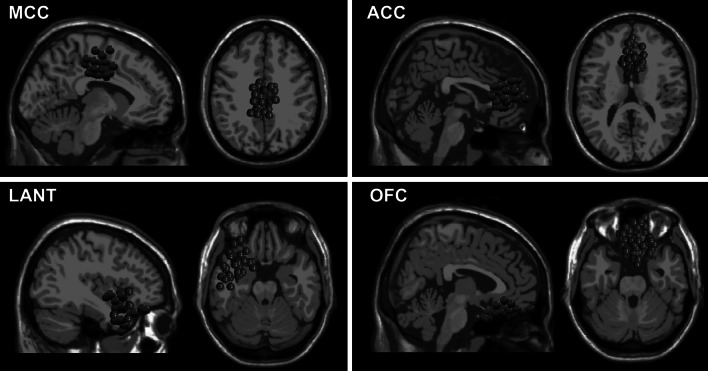
Location of clusters significantly associated with VAD dimensions with the constituting independent components

The clustering procedure of the EEG-independent components revealed nine brain-related, cortical clusters. Their location in the standardized Talairach space is depicted in Table 3. Four of them revealed significant regression effects. For the MCC cluster, beta power activation was significantly predicted by negative PANAS-X scales of low dominance (*R* = 0.50; *F*(2,26) = 9.43; *p* = 0.005; *p*_adj_ = 0.045). The ACC cluster showed significant relationships with the PANAS-X scales of negative valence and low arousal (*R* = 0.41; *F*(1,33) = 6.97; *p* = 0.013; *p*_adj_ = 0.039). In the case of the LATL cluster, the best fit of the regression model (*R* = 0.53; *F*(2,23) = 8.06; *p* = 0.002; *p*_adj_ = 0.018) was found for negative valence emotions of both high (*β* = 0.47; *t*(23) = 2.45; *p* = 0.03) and low arousal (*β* = 0.52; *t*(23) = 3.07; *p* = 0.01). Finally, for the OFC cluster, the significant model included the category of negative scales with high arousal (*R* = 0.46; *F*(1,34) = 8.91; *p* = 0.005; *p*_adj_ = 0.022). Figure 2 shows the locations of clusters which revealed significant correspondence with the VAD dimensions. Results of additional correlational analysis between clusters’ beta power and discrete PANAS-X scales are shown in Table 4. The regression results were verified using the leave-one-out cross-validation procedure (LOOCV), which revealed relatively high accuracies of the models (MCC: 72.6 %, LATL: 84.7 %, ACC: 75.9 %, OFC: 77.4 %).

**Table 3:** The locations of revealed clusters

Cluster no.	Location	Cluster centroid Talairach coordinates		
		*x*	*y*	*z*
1	Middle cingulate cortex (MCC)	−19	−11	34
2	Right anterior temporal lobe (RATL)	39	3	−23
3	Anterior cinglulate cortex (ACC)	4	10	8
4	Right temporoparietal junction (RTPJ)	31	−51	21
5	Posterior cingulate cortex (PCC)	−7	−33	28
6	Right posterior temporal lobe (RPTL)	30	−67	13
7	Right parietal lobe	43	−37	29
8	Left anterior temporal lobe (LATL)	−36	8	−18
9	Orbitofrontal cortex (OFC)	7	30	−22

**Table 4:** Significant correlations between PANAS-X scales and beta power in selected IC clusters

	Hostility	Guilt	Fear	Sadness	Shyness	Fatigue	Surprise	Calmness	Attentive	Self-assurance	Joviality
MCC			0.39*		0.34*	0.36*		−0.38*			
RATL	0.43**										
ACC				0.37*	0.31*	0.33*					
RPTL											0.33*
PCC											0.54**
LATL	0.31*	0.45**	0.43*	0.44**	0.43**					−0.36*	
OFC	0.35*		0.27*								

* *p* < 0.05; ** *p* < 0.01

## Discussion

The current study explored whether the VAD (*valence*, *arousal*, and *dominance*) dimensional model of emotion can reflect the neural basis of subjectively experienced affect in a clearer and more understandable way than the locationist approach based on discrete emotions.

Three of the brain clusters (RATL, RPTL, and PCC) were related to only one discrete emotion (however, not in a unique way), while another four (MCC, ACC, LATL, and OFC) showed correlations with more than one emotion. When analysing relationships between discrete PANAS-X emotions and the activity of brain clusters, six of the scales (hostility, fear, sadness, shyness, fatigue, and joviality) were associated with more than one cluster, while another three (guilt, calmness, and self-assurance) were linked to a single one. No significant effects were found for surprise and attentiveness. Although these screening results should be treated with caution, it was shown that the relationships between discrete emotions and brain clusters are far from being clear. In this respect, the results resemble numerous studies (mostly from the fMRI domain), where co-variation effects between emotions and focal brain activations were found; however, many of them were difficult to replicate (Kober et al. 2008; Lindquist et al. 2012). These observations are contrary to the basic postulate of the locationist approach with regard to the uniqueness and specificity of brain activations for different emotions.

Analysis of the self-reports expressed in terms of the VAD dimensions revealed that the activity in four out of nine brain clusters was related to the current emotional state. The cluster located in the ACC was associated with emotions of negative valence and low arousal. Indeed, hyperactivation of this structure is often reported in individuals suffering from depressed mood or chronic pain (Nemoto et al. 2007; Yoshimura et al. 2010; Vytal and Hamann 2010; Etkin et al. 2011). The ACC is also thought to be a part of the network associated directly with the subjective experience of sadness (Damasio et al. 2000; Phan et al. 2002; Habel et al. 2005) and the affective aspect of pain (Singer et al. 2004; Medford and Critchley 2010). Our results fully support these data and, moreover, emphasize the common experiential quality related to the activation of the ACC: negative valence and low arousal.

Contrarily, the OFC cluster turned out to be associated with negative and highly arousing emotions. This is in line with some studies where activation of this structure was associated with emotions of fear, disgust, or anger (Phan et al. 2002; Murphy et al. 2003; Lindquist et al. 2012) but in contrast to others, where the OFC was also related to positive emotions (Kirkland and Cunningham 2011; Wyczesany et al. 2014). The orbitofrontal areas are well known to be crucial for many emotional functions, including appraisal and assigning a motivational value to stimuli for both positive and negative emotions. These areas are also possibly linked with the experience of affective value of both concrete and abstract stimuli (O’Doherty et al. 2003; Rolls and Grabenhorst 2008; Carvajal et al. 2013). It has also been shown that distinct OFC regions can be associated with either positive or negative emotions (Kringelbach 2005). However, it is not clear to what extent these observations are associated with affective experience per se and are not confounded by emotional perception or motivational learning. The fact that positive emotions are linked mostly to the medial OFC, which is more difficult to reach by surface recording, can explain the lack of positive valence effects in our data. The spatial resolution of EEG, however, does not allow for such precise inferences about source localization, and more data are needed to clarify this issue.

Interestingly, within negative emotions, there was a double dissociation observed, where the ACC was related only to low arousing, while the OFC only to highly arousing emotions (this is also visible in the results of correlations with discrete PANAS-X scales). This suggests that these two categories of emotions involve at least partly separate circuits.

The cluster located in the MCC was, interestingly, related to low dominance emotions of negative valence. In the literature, the activity of this area is usually attributed to pain, disgust, fear, or sadness (Vogt 2005; Colibazzi et al. 2010; Stevens et al. 2011; Lindquist et al. 2012), which are indeed unpleasant emotions characterized by a low sense of control. On the other hand, an analysis by Anderson et al. (2013) based on 2603 neuroimaging experiments in different task domains, showed that the MCC region (labelled there as dACC) is strongly associated with the emotion of disgust, moderately with fear, happiness and sadness, and not related to anger. Although these findings partly support our observations, they also mention some positive states. When taking a deeper look at the intermediate steps of the regression analysis, the low dominance negative and positive emotions were also found to significantly predict the MCC activity. However, finally, the model with a single predictor (i.e. negative valence and low dominance) was chosen as the optimal one, bearing most predictive power. Hence, although this result ultimately points exclusively to negative, low dominance emotions, some contribution of positive states of low dominance cannot be ruled out. This issue certainly requires more data in order to be entirely clarified, but the role of dominance as an important discrimination factor explaining the activity of the MCC is supported.

Lastly, the LATL cluster was linked in our data with all types of negative emotions. The literature data link this region with experiencing sadness, anxiety, fear, and anger (Gloor et al. 1982; George et al. 1995). A review by Wong and Gallate (2012) mentions that this area is activated during a wide range of tasks including: watching sad, angry or disgusting movies, and recalling events related to emotions of anger, fear, sadness, or anxiety (effects were specific for the left or bilateral hemisphere). Another review by Lindquist et al. (2012) showed that experience of anger is most likely to activate the LATL (compared with other basic emotions). Generally, many negative emotions, irrespective of arousal or dominance, are confirmed to activate this area, and these characteristics are possibly related to the nearby limbic area (Olson et al. 2007). It is also clearly seen that the LATL is not specific to any single discrete emotion. However, it should be noted that a few reviewed experiments showed some activations in the LATL during positive emotions as well. Hence, it is still possible that negative valence cannot fully explain the activation of this region, and that this region is involved in mediating emotional processes of both negative and positive valence. It can also be explained by association with the left amygdala, which is recruited during processing of emotionally arousing content, irrespective of valence (Hamann and Mao 2002).

An alternative perspective for understanding the role of the LATL is brought by recent data from Lindquist et al. (2014) who argues that this area associated with semantic memory is crucial for integrating affective perception with conceptual knowledge on discrete emotions and their meaning. This interpretation comes from the observation of patients with LATL lesions and their impaired perception of discrete emotions but not basic affective states (valence dimension). On the basis of the constructionist approach, which assumes the common neural ground of different aspects of emotional processes, we could suggest that the proposed role of the LATL area also applies to emotional experience. Moreover, the fact that perception of basic affective dimensions (valence) was preserved may suggest that the description of emotional experience on the VAD dimensions is more basic and associated with core affect, while discrete emotions are more dependent on socialization and conceptualization processes.

The question arises as to why most of our observations relate to negative emotions, while the literature data regarding positive affect (especially in the case of the LATL and OFC clusters) were not confirmed. However, it is often observed that effects of positive emotions, especially in laboratory conditions, tend to be weaker than those visible in negative states, which was the case in our study (George et al. 1995). We can speculate that our experimental design, with no explicit emotion induction, did not differentiate subjects sufficiently to provide enough power for statistical tests in the case of the positive items. It is also possible that positive states—which are phenomenologically less specific^2^—are more difficult to investigate. Some data suggest that positive emotions can be associated with opposite activations within nearby brain areas (Damasio et al. 2000), which could cancel each other out in an EEG recording characterized by low spatial resolution. Certainly, this apparent dominance of negative emotions in predicting brain activity patterns requires furthers studies. Moreover, other measurement conditions, including active task and mood manipulations, need to be considered in future research in order to identify the potential bias of passive rest conditions (Wyczesany et al. 2011).

Taken together, experience of negatively valenced emotions was associated with activations of all considered clusters; however, they differed regarding either the arousal (ACC, OFC, and LATL) or the dominance (MCC) dimension. This shows that self-description of emotional experience using VAD dimensions can reflect real brain activity, which adds support to recent fMRI data (Nielen et al. 2009; Baucom et al. 2012). The analysis based on higher level, i.e. more general, dimensions turned out to introduce more clear and ordered relationships between self-report and brain activity than observed in the case of discrete emotions. This result favours the constructionist view, which assumes that our emotions are not based on separate and specific brain “processors”, but are rather constructed from more general dimensions by distributed neural networks of interconnected structures. Their activity in different configurations could “produce” emotions, which are then labelled as discrete entities. Thus, emotions which are located close to each other in the VAD space are supposed to show relatively similar patterns of brain activations. This is also supported in our data, where similar emotions gathered together in VAD categories could be associated with activations of specific clusters. Concluding, our results show that self-report expressed in terms of VAD dimensions can explain a significant part of the variability observed in selected brain areas.

Finally, some limitations and future directions should be mentioned. As our conclusions were drawn on the basis of a passive, no-task procedure, it would be recommended to extend similar analysis to other experimental conditions. Indeed, the majority of studies on emotions used some kind of affect induction, among which standardized visual pictures are most common (Lang et al. 1999; Keil et al. 2002). As the processes of emotional perception and emotional experience are tightly bound, comparison of results in conditions of evoked affect remains an important issue. One should also be aware that gender differences may play a role in emotional processes (Sabatinelli et al. 2004). We also note that the stepwise regression applied here is not hypothesis driven and thus increases the risk of type I errors. The exploratory nature of this study can justify this approach and additional precautions (multiple comparison correction, cross-validation) to minimize possible chance findings. Validation of revealed ROIs with the NIRS technique would also be beneficial to allow for both precise localization and (unlike the fMRI) relatively natural measurement conditions.

## Footnotes

1 The low number of discrete emotions prevented an all-in-one 2 × 2 × 2 analysis. Valence as a core dimension for emotional experience was then used in both regressions.

2 The list of five basic emotions includes happiness, sadness, fear, anger, and disgust, where only the first one is positive. This negative bias is also visible in the number of emotion labels in many languages (Rozin et al. 2010).
